# Colonoscopic Diagnosis of Postoperative Gastrointestinal Bleeding in Patients With Hirschsprung's Disease

**DOI:** 10.3389/fped.2021.672767

**Published:** 2021-06-28

**Authors:** Jixin Yang, Tianqi Zhu, Xiaojuan Wu, Mingfa Wei, Guo Wang, Jiexiong Feng

**Affiliations:** Department of Pediatric Surgery, Tongji Hospital, Huazhong University of Science and Technology, Wuhan, China

**Keywords:** Hirschsprung disease, gastrointestinal bleeding, enterocolitis, colonoscopy, hematochezia

## Abstract

**Aim:** Postoperative lower gastrointestinal bleeding in children with Hirschsprung's Disease (HSCR) is a non-specific symptom, which may be caused by various etiologies. Our current study aims to utilize colonoscopy to diagnose the causes of postoperative hematochezia and to analyze its feasibility, accuracy, and safety.

**Methods:** Twenty-four patients with HSCR with postoperative lower gastrointestinal bleeding or occult blood in the stool were enrolled in this study. The postoperative onset duration, age at examination, accompanied anomalies were recorded. After bowel preparation, all patients underwent colonoscopy. According to visual findings, mucosal biopsy was performed, followed by pathological diagnosis. Further treatment was determined according to the visual findings and pathological diagnosis. All patients were followed up for 6 months including therapeutic outcomes and recurrence of symptoms.

**Results:** The mean onset duration was (221.3 ± 216.8) days postoperatively (ranging from 25 to 768 days). The mean age at examination was (41.0 ± 29.4) months. There was no significant difference in the onset days among each group (all, *p* > 0.05). Based on visual and pathological findings, there were 11 cases of HSCR associated enterocolitis (HAEC), 4 cases of anastomotic leakage, 7 cases of anastomotic inflammation, 1 case of juvenile polyp, and 1 case of inflammatory pseudopolyp. Intraluminal saline irrigation, thrombin treatment or colorectal polyp electrocision was performed according to intraoperative diagnosis. Patients with HEAC and anastomotic inflammation underwent antibiotics therapy and colorectal irrigation. Patients with leakage underwent reoperation. The highest incidence of accompanied symptoms of diarrhea existed in HEAC group (*p* = 0.02) and fever in leakage group (*p* = 0.02), respectively. No perforation or aggravated bleeding occurs in any patients. All patients gained uneventful recovery during follow-up period.

**Conclusions:** Colonoscopy is a safe, accurate and timely examination for HSCR patients with postoperative lower gastrointestinal bleeding. The visual findings and biopsy may provide accurate diagnosis and guide treatment for this subset of patients.

## Introduction

Although the symptom of rectal bleeding was usually considered to be non-specific (1), when patients with Hirschsprung's disease (HSCR) experienced hematochezia or occult stool postoperatively, great attention should be paid to the postoperative HSCR associated enterocolitis (HAEC) (1), anastomotic lesions (2), leakage (3) or other complications. Timely diagnosis and treatment can avoid further progress of these complications. For most of patients with hematochezia or positive occult blood, colonoscopy was reported to be a safe, crucial, and accurate examination (4). It help doctors make macroscopical diagnosis through direct vision and histological diagnosis by mucosal biopsy, therefore, it helps pediatric surgeons carry out corresponding treatments (5). Although some sporadic reports showed the application of colonoscopy in diagnosing HAEC or other minor lower gastrointestinal bleeding, till now, there is no report on colonoscopic diagnosis for the group of HSCR patients experiencing postoperative gastrointestinal hematochezia presents or occult blood, nor there is any consensus of the technique and appropriate case target selection.

This retrospective study reported a group of children with HSCR experiencing hematochezia or fecal occult blood after pull-through procedures. All patients underwent colonoscopy and received endoscopic mucosal biopsy. According to specific diagnosis, corresponding treatments or reoperation were chosen by doctors. The importance and safety of colonoscopy in this subset of patients were evaluated, analyzed and discussed.

## Patients and Methods

From October 1st 2016 to June 30th 2020, a total of 301 cases of children with HSCR in our department received pull-through operations. The length of resected bowels was evaluated preoperatively based on results of barium enema and determined intraoperatively by rapid frozen section followed by histological examinations.

Among them, 24 cases had recurrent hematochezia or occult blood after stool examination. All patients had more than or equal to 2 times of onsets. The mean postoperative onset duration, presence of clotting abnormalities and associated anomalies including diarrhea, fever, and abdominal distension were retrieved from the clinical database.

Before colonoscopy, electrocardiogram, blood routine examination, and coagulation test were performed. Colonoscopy was performed in the digestive endoscopy room under intravenous anesthesia. The digestive tract was prepared 1 day before operation, and saline irrigation was performed once using a 16Fr silicone anal tube. Patients need to be fasting for 2 h before colonoscopy. Intravenous Cephalosporins (100 mg/Kg) twice a day and metronidazole (10 mg/Kg) once a day were given. Nasogastric tube was intubated in patients with abdominal distension. According to the macroscopic findings, mucosal biopsy, colorectal polyp electrocision, or intraluminal saline or thrombin treatment were performed. The corresponding further treatment was determined according to the visual findings of colonoscopy and pathological diagnosis. Patients with HAEC were treated with abovementioned antibiotics and colorectal saline irrigation until relief of all symptoms, and disappearance of red blood cell or white blood cell in stool. Patients with anastomotic leakage underwent enterostomy after colonoscopy. Patients with unabsorbed thread head and anastomotic inflammation were given Cephalosporins orally for a week, and we did not perform saline irrigation unless occult blood was persistent. Under the colonoscope, local, or extensive submucosal congestion, mucosal edema, mucosal ulcer accompanied with or without intraluminal bleeding were considered to be the macroscopic signs of HAEC. According to Elhalaby et al.'s (6) histological criteria, pathological diagnosis of HAEC was made.

During examination, patients took the right lateral position, and the surgeons stood by the left hand side of patients. When operating the colonoscope, as long as the view was clear, we did not frequently inflate or flush saline to ensure low pressure in the bowel cavity.

All patients were followed up for 6 months, including outcomes and recurrence of hematochezia. The model of digestive endoscopy system was vp-4450hd (Fujinon, Japan), and the model of colonoscopy was ec-530wm (Fujinon, Japan). Continuous data were analyzed by *t*-test after analysis of variance, and category data were compared by Fisher's exact test. *P* < 0.05 showed statistical difference.

## Results

In the 24 patients, including 13 males and 11 females, the average onset of gastrointestinal bleeding was (221.3 ± 216.8) days postoperatively (ranging from 25 to 768 days). The average age at examination was (41.0 ± 29.4) months. There was no significant difference in the onset days among the groups (*P* > 0.05). There were 12 cases of hematochezia and 12 cases of occult blood. Ten cases were accompanied with diarrhea, 11 with fever, and 4 with abdominal distension which were relieved after antibiotics treatment and colorectal saline irrigation.

Sixteen cases of short segment HSCR were treated with left hemicolectomy, five cases of long segment HSCR, and three cases of HSCR complicated with intestinal neuronal dysplasia were treated with subtotal colectomy. Eighteen cases were treated with heart-shaped anastomosis (7, 8), four cases with Soave anastomosis, one case with Rehbein anastomosis, and one case with Duhamel anastomosis. Overall, only the average prothrombin time at admission (15.4 ± 1.0 s) slightly increased compared to the reference value (11.5–14.0 s). The average values of other parameters including prothrombin activity, international normalized ratio, fibrinogen, activated partial thromboplastin time, and thrombin time were all within the normal range. Based on visual and pathological findings, there were 11 cases of HAEC, four cases of anastomotic leakage, seven cases of anastomotic inflammation, one case of juvenile polyp, and one case of inflammatory pseudopolyp. No lesion was observed in ileocecum region. None of the patients had perforation, aggravated bleeding or other endoscopy related complications after examination. There was no significant difference in the ratio of hematochezia to occult blood between patients with HAEC and other patients (all, *P* > 0.05). The incidence of diarrhea in HAEC group was significantly higher than that in anastomotic inflammation group (9/11 vs. 1/7, ^*^P = 0.02), but there was no significant difference compared with anastomotic leakage group or polyp group (both, *P* > 0.05). The incidence of fever in HAEC group was significantly lower than that in anastomotic leakage group (2/11 vs. 4/0, ^**^P = 0.02), but there was no significant difference compared with other groups (both, *P* > 0.05). There was no significant difference in the incidence of abdominal distension among the groups (*P* > 0.05; Table 1).

**Table 1:** Overview of pathology, symptoms, and signs.

**Pathological changes**	**Cases**	**Mean postoperative onset duration (days)**	**Symptoms and signs**			
			**Hematochezia /fecal occult blood**	**Diarrhea**	**Fever**	**Abdominal distention**
Hirschsprung's disease associated enterocolitis	11	216.8 ± 261.7	4/7	9^*^	2^**^	3
Anastomotic leakage	4	173.5 ± 61.5	1/3	1	4	1
Anastomotic inflammation	7	241.3 ± 235.3	5/2	1	4	0
Polyp	2	271.5 ± 188.8	2/0	0	1	0

* *P = 0.02, compared to anastomotic inflammation group*.

** *P = 0.02, compared to anastomotic leakage group*.

**Figure 1:**
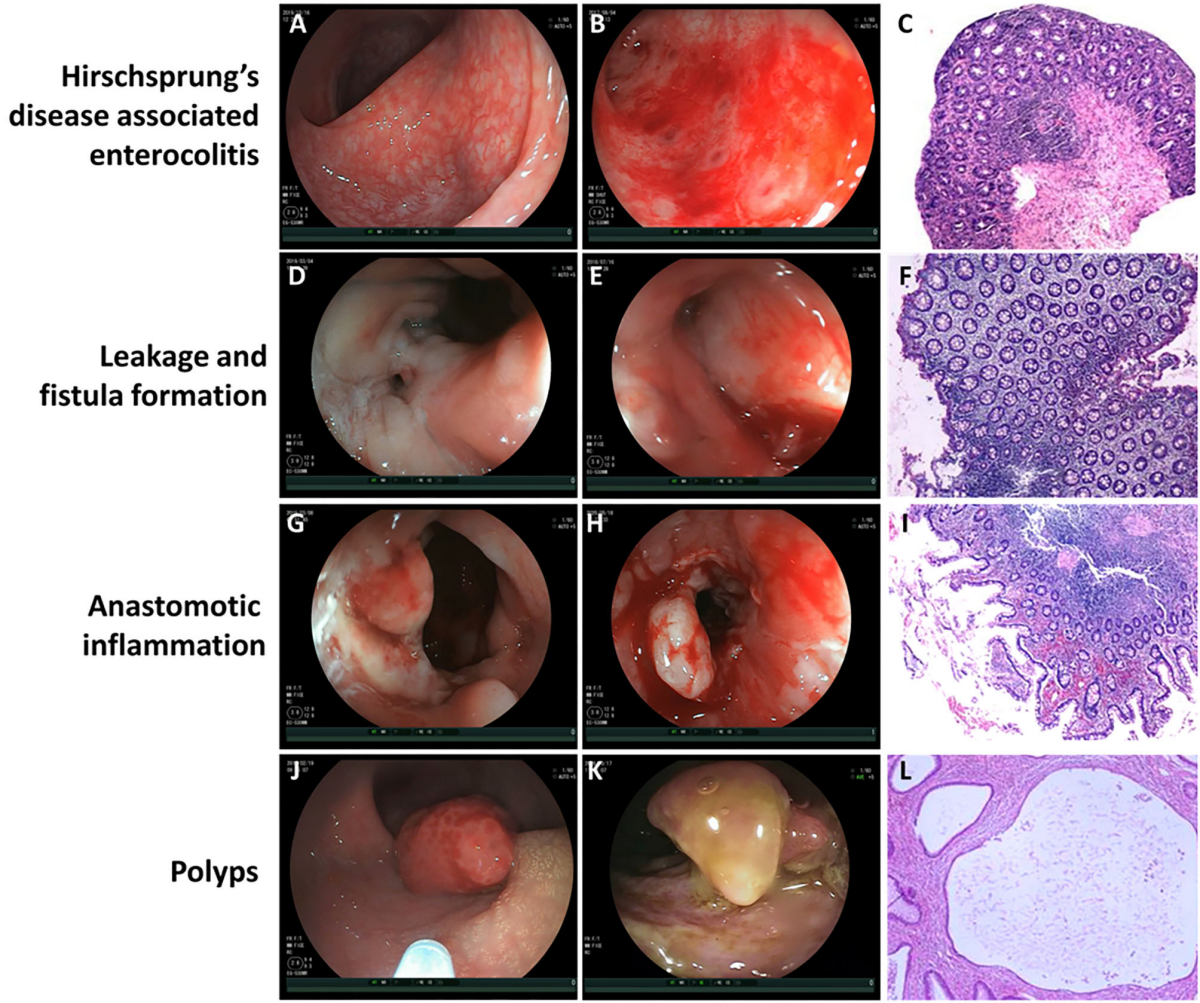
The visual finding of colonoscopy and pathological changes under microscope. **(A)** Extensive congestion and mucosal edema of the colon and neorectum in patients with Hirschsprung's disease associated enterocolitis (HEAC). **(B)** Mucosal ulceration and bleeding with mucosal annular protrusion and mucosal ulcer formation in patients with HAEC. **(C)** Extensive chronic inflammation in colonic and rectal mucosal tissues with mucoid and inflammatory effusion under microscope. Magnification ×100. **(D)** Fistula formation near the anastomotic leakage. **(E)** Mucosal edema and congestion around the leakage. **(F)** Chronic inflammatory cell infiltration and focal erosion in the intestinal mucosa under microscope. Magnification ×100. **(G)** Mucosal erosion, edema, and bleeding near the anastomotic site in patients with anastomotic inflammation. **(H)** Mucosa was locally uplifted and brittle, and bleeding was obvious after scratching in patients with anastomotic inflammation. **(I)** Chronic infiltration of inflammatory cells in the mucosa near the anastomotic site, accompanied by focal hemorrhage and extensive erosion under microscope. Magnification ×100. **(J)** Juvenile polyp. **(K)** Inflammatory pseudopolyp. **(L)** Focal areas of mucosal hyperplasia with cystic dilatation of the crypts and accumulation of mucus within the cysts under microscope (Juvenile polyp). Magnification ×100.

According to the severity of clinical episodes of HAEC (9), nine patients were scored as Grade 1 and the other 2 were scored as Grade 2. In 11 children with HAEC, extensive congestion and mucosal edema of the colon and neorectum were observed under the colonoscopy (Figure 1A). Some patients had mucosal ulceration and bleeding with mucosal annular protrusion and mucosal ulcer formation (Figure 1B). Some children had remote hemorrhage in the colorectal cavity (Supplementary Video 1). Mucosal biopsy showed that eight cases had extensive chronic inflammation in colonic and rectal mucosal tissues, where some of the mucosa disappeared with mucoid and inflammatory effusion (Figure 1C). Two cases were accompanied by infiltration of a small amount of foam cells, lymphoid cells, plasma cells and formation of lymphoid follicles. One case was accompanied by lymphoid hyperplasia in the lamina propria, lymphoid follicles, and scattered eosinophilic infiltration. After completion of the examination, 11 children were treated with intravenous antibiotics and colorectal saline irrigation, and all recovered and discharged. Two cases had recurrent HAEC during a followed-up period of 6 months after discharge.

There were four cases of anastomotic leakage, including one case of short segment HSCR with intestinal neuronal dysplasia (heart-shaped anastomosis), two cases of long segment HSCR (one case of Rehbein anastomosis, one case of heart-shaped anastomosis), and one case of short segment HSCR (Soave anastomosis). Colonoscopy showed fistula formation near the anastomotic leakage (Figure 1D), mucosal edema and congestion around the leakage (Figure 1E). In one case, a sacrococcygeal external fistula was formed. Diluted methylene blue injection injected from the external fistula was observed flowing out of the internal fistula (Supplementary Video 2). Mucosal biopsy near the internal fistula showed chronic inflammatory cell infiltration and focal erosion in the intestinal mucosa (Figure 1F). All of the four patients underwent enterostomy after colonoscopy. Three months later, the fistulectomy, re-pull-through, and Soave anastomosis were performed again. Two months later, the stoma was closed. All the patients recovered well during follow-up period.

In six children with anastomotic inflammation, mucosal erosion, edema, and bleeding near the anastomotic site were observed under colonoscope (Figure 1G). Part of the mucosa was locally uplifted and brittle, and bleeding was obvious after scratching (Figure 1H). After scratching the uplifted and edematous mucosa, unabsorbed thread head was observed in some cases, which was removed under colonoscope. Anastomotic stenosis could be seen in 1 patient. Duhamel's procedure was performed in one of them, and the formation of blind rectal pouch was observed (Supplementary Video 3). Macroscopically, the mucosa on the septum after Duhamel's procedure was swollen and congestive, with sporadic punctate hemorrhage. Mucosal biopsy showed chronic infiltration of inflammatory cells in the mucosa near the anastomotic site, accompanied by focal hemorrhage and extensive erosion (Figure 1I). After 6 months of follow-up, symptoms disappeared in 4 cases after antibiotics treatment and saline irrigation. In 1 case accompanied with anastomotic stenosis, after antibiotics therapy and saline irrigation for 1 week, no recurrence of hematochezia was observed. Three weeks later, anal dilation was performed for 2 months.

Two children were diagnosed as polyps by colonoscopy. One patient had a polyp with a diameter of 8 mm at ~30 cm from the anastomotic site, which was diagnosed as juvenile polyp by pathological examination (Figure 1J). In the other case, a polyp with a diameter of about 1.5 cm was found at the anastomotic site, with the width of about 4 mm at the bottom (Figure 1K). These 2 cases were diagnosed as juvenile polyp (Figure 1L) and inflammatory pseudopolyp, respectively. After 6 months of follow-up, there was no recurrent hematochezia or occult blood in stool in the two cases.

## Discussion

In 1998, Balkan et al. (10) reported sigmoidoscopy in minor lower gastrointestinal bleeding in 2 patients with HAEC. Although Balkan's report is the first one focusing on application of endoscopy for HAEC, our report focused on lower digestive endoscopy for hemorrhagic issues following surgery for HSCR, with different targeted subset. The postoperative hematochezia needs to be identified by pediatric surgeons in time, because the most common cause may usually be HAEC, anastomotic inflammation, anastomotic leakage, and polyps, as shown in our current study. In our study, hematochezia or occult blood occurs at 25 days to more than 2 years after primary pull-through operation. Early diagnosis could avoid poor prognoses such as septic shock, perforation, acute/chronic anemia and hemolysis shock in this subset of patients (11). Pediatric surgeons were usually used to diagnosing HAEC from clinical manifestations and laboratory examinations (1), however hematochezia of a part of patients were caused by local anastomotic site. The two groups of patients were sometimes difficult to differentiate, meanwhile their treatments are quite different (12). When anastomotic leakage occurs, it usually needs staged reoperation (13), while inflammatory diseases usually need conservative therapy (14). Therefore, timely and accurate diagnosis is of great significance for determining strategies of treatment for postoperative hematochezia with various etiologies.

Colonoscopy has been widely used in the diagnosis and treatment of lower gastrointestinal bleeding in children (5). Total colonoscopy is now very common for patients over 1 year old (15). However, in our study, this subset of patients has distinctive features, and the operating technique of colonoscopy is different from the reports when diagnosing lower gastrointestinal bleeding. Firstly, the children have already undergone pull-through endorectal anastomosis. According to the length of the removed bowel segment, the length and shape of the residual bowel after operation are different from unoperated children. For children with short segment HSCR, the left hemicolectomy is usually performed. The anatomical position has changed after the splenic flexure is dissociated, so that the angle of bowel is sharper when entering the transverse colon. Especially when postoperative HAEC occurred, the mucosal becomes swollen and fragile, and the intestinal wall becomes weak due to long-term explosive diarrhea and overproduction of aerogenic bacteria (6). Therefore, great attention should be paid to the total colonoscopy to avoid perforation or mucosal injury. For children with long segment HSCR and IND, subtotal colectomy is often performed, and ileocecum can be observed easily by colonoscopy. Secondly, through our observation, there are very few lesions only located in ileocecum in children with postoperative gastrointestinal bleeding. Based on the abovementioned points of view, we think that it is unnecessary for all children to undergo total colonoscopy, except for those who had no obvious lesions near the anastomotic site and needed to be examined until the ileocecum. For example, in one patient of our study, a juvenile polyp was found at the transverse colon at 30 cm proximally to the anastomotic site. Thirdly, we consider that it is unnecessary to fully inflate the colon to expose the space during colonoscopy, because the operation of biopsy does not need to fully dilate the colon, and excessive inflation may increase the risk of perforation in children with HAEC. During colonoscopy, we used low pressure inflation, therefore, none of the children in this study had perforation. Our practice confirmed the safety of colonoscopy for HSCR patients postoperatively. Meanwhile, we consider that patients with extreme abdominal distension or peritonitis should not undergo colonoscopy unless the symptoms were relieved after conservative treatment, otherwise the risk of perforation may be very high (1).

Besides safety issues, through our practice, we also consider that colonoscopy is an efficient and accurate examination for the diagnosis of postoperative hematochezia or fecal occult blood. One of the most important reasons is that obtaining tissue samples at the suspicious lesions for pathological diagnosis is the most direct evidence and most accurate scientific fundament, which provides evidence for determining clinical treatment (16). We found that the pathological characteristics of HAEC were acute or chronic inflammatory changes with lymphoid tissue hyperplasia and lymphoid follicle formation in lamina propria, and most of the bleeding may be caused by mechanical stress stimulation when feces pass through and destruction of mucosal barrier after toxin absorption (17). In those with anastomotic inflammation, biopsy of inflammatory and ulcerative surface near the anastomotic site indicated chronic inflammatory cell infiltration, which was accompanied by focal bleeding and extensive erosion. It is often due to mechanical stimulation of stool caused by poor anastomosis techniques, delayed suture reaction, suture shedding, and anastomotic stenosis, resulting in long-term inflammatory stimulation of the anastomotic site and adjacent mucosal tissue, which lead to ulceration and bleeding (18). If the anastomotic inflammation does not heal in time, part of the anastomotic site may form inflammatory pseudopolyps secondary to repeated bouts of intense inflammation (19), leading to periodic bleeding which is similar to the symptoms of juvenile polyps. For this part of patients with inflammatory hemorrhagic changes, saline, thrombin, and other drugs for local irrigation treatment were applied directly under colonoscopy. What is more, colonoscopy provides us a timely and objective basis for the reoperation of children with leakage or blind rectal pouch formation after Duhamel's procedure, so as to avoid delaying the opportunity of surgical treatment (20). Finally, through retrospective analysis, we found that the incidence of diarrhea in HAEC group was significantly higher than that in anastomotic leakage group, and the incidence of fever was the highest in patients with leakage, which were both consistent with a previous literature (21).

We have to recognize that there were some limitations in this study. Since our department started to set up independent children's digestive endoscopy center in 2016, the number of cases collected was relatively small, accounting for 7.97% of all children with HSCR undergoing operations in the same period. By using our technique and preoperative preparation, although there was no complication in colonoscopy and its accuracy was 100%, it still needs to further expand the sample size to fully evaluate the safety and accuracy of colonoscopy in children with HSCR after operation, and set up excluding criteria to avoid site injuries. Additionally, the colonoscopy does not apply to examining bleeding in short term after surgery, because we worried that it may affect the healing of the anastomosis. Thus, there is selection bias in this study. Lastly, the retrospective assessment limits the interpretation of our results, and a prospective study is expected in future.

## Conclusion

In summary, for HSCR patients with postoperative gastrointestinal bleeding, colonoscopy is safe and effective if appropriate cases were selected carefully. Colonoscopy can accurately provide location of the lesions, clarify the characteristics and pathological changes, and therefore provide timely and reliable basis for corresponding treatment.

## Data Availability Statement

The original contributions presented in the study are included in the article/Supplementary Material, further inquiries can be directed to the corresponding author/s.

## Ethics Statement

The studies involving human participants were reviewed and approved by the Institutional Review Board of Tongji Hospital, Tongji Medical College, Huazhong University of Science and Technology. Written informed consent to participate in this study was provided by the participants' legal guardian/next of kin.

## Author Contributions

JY designed and executed the study, performed statistical analyses, drafted the figures and tables, and wrote the consecutive versions of the manuscript. TZ, XW, MW, and GW performed statistical analyses, critically reviewed and commented all versions, interpreted findings, and contributed to the discussion. JF conceptualized and designed the study, coordinated and supervised data collection, and critically reviewed the manuscript for important intellectual content. All authors agree to be accountable for the content of the work.

## Conflict of Interest

The authors declare that the research was conducted in the absence of any commercial or financial relationships that could be construed as a potential conflict of interest.

## Supplementary Material

Supplementary Video 1 Remote hemorrhage in the colorectal cavity.

Supplementary Video 2 Diluted methylene blue injection injected from the external fistula was observed flowing out of the internal fistula.

Supplementary Video 3 Blind rectal pouch.
